# Effects of storage time on DNA profiling success from archived latent fingerprint samples using an optimised workflow

**DOI:** 10.1080/20961790.2020.1792079

**Published:** 2020-08-03

**Authors:** Sydney I. Menchhoff, April D. Solomon, Jordan O. Cox, Madison E. Hytinen, Marilyn T. Miller, Tracey Dawson Cruz

**Affiliations:** a Department of Forensic Science, Virginia Commonwealth University, Richmond, Virginia, USA; b Jefferson Parish Sheriff’s Office Regional DNA Laboratory, Harvey, Louisiana, USA

**Keywords:** Forensic sciences, forensic DNA, low template DNA, touch DNA, aged samples, archived latent fingerprints, optimised workflow

## Abstract

Due to recent improvements in forensic DNA testing kit sensitivity, there has been an increased demand in the criminal justice community to revisit past convictions or cold cases. Some of these cases have little biological evidence other than touch DNA in the form of archived latent fingerprint lift cards. In this study, a previously developed optimised workflow for this sample type was tested on aged fingerprints to determine if improved short tandem repeat (STR) profiles could be obtained. Two-year-old samples processed with the optimised workflow produced an average of approximately five more STR alleles per profile over the traditional method. The optimised workflow also produced detectable alleles in samples aged out to 28 years. Of the methods tested, the optimised workflow resulted in the most informative profiles from evidence samples more representative of the forensic need. This workflow is recommended for use with archived latent fingerprint samples, regardless of the archival time.

**Key points**
- The use of the optimised workflow on aged archived latent fingerprint (ALFP) lift card samples (aged 2–28 years) improves the number of STR alleles recovered, providing more discriminatory STR profiles than those processed using the traditional workflow.
- Interpretable STR alleles can be detected from ALFP lift card samples stored as long as 28 years when the optimised procedures are followed.
- The use of individual laboratory-sterilised tools for sample preparation and the addition of a re-purification step with Centri-Sep columns in the recommended optimised workflow seem to limit the ability to detect low-level secondary DNA sources.

## Introduction

Since its first exoneration in 1989, the Innocence Project has used DNA testing to overturn convictions of more than 350 wrongfully incarcerated people, many of whom were on death row [1]. DNA analysis has become a routine part of a criminal investigation in modern day forensic testing, as well as the re-testing of old or cold cases, but this was not always the case. While short tandem repeat (STR) analysis can greatly increase the discriminatory power compared to preceding analytical techniques, this method only became common practice in the early 2000s [2]. Thus, previous convictions were often based on less discriminating DNA tests or other, less reliable, non-DNA based methods of identification. Additionally, cold cases that were not solvable with older techniques now can be revisited. As the Innocence Project has shown, there is an increased demand to re-evaluate old cases and re-examine the evidence, often in the form of conducting DNA tests that were not previously possible [1]. In many of these cases, with the absence of body fluids, the only viable DNA evidence may be found in the form of archived latent fingerprint (ALFP) lift cards.

Latent fingerprints often contain touch DNA, which comes from shed epithelial cells. On average, humans shed approximately 400 000 epithelial cells a day [3]. When part of the body, such as a fingertip, comes into contact with a surface, it will leave behind some of these cells, along with secreted sweat and oils [4]. While many of the cells that are shed from the outer layer of skin are enucleated, there is often fragmented DNA present among the keratinised cells [5]. Additionally, as the sweat and oils pass through glands, they collect epithelial cells and cell-free DNA which are also deposited alongside the fragmented DNA [4,6]. ALFPs are fingerprints that are typically visually enhanced by chemical or physical means, tape-lifted, then placed on a paper lift card [7,8]. This collection process leaves the DNA, oils, and sweat “sandwiched” between the tape and paper, enabling an investigator to peel the tape away and collect DNA from the assembly [9]. This is contrary to modern-day casework, in which fingerprints or touch evidence that are deemed eligible for DNA analysis are usually directly swabbed at the scene after their patterns are documented. Fortunately, ALFP lift cards may retain DNA for an extended period and could serve as an alternative investigative tool for both older and recent cases that lacked useful results from friction ridge analysis due to the presence of smudges or smears. Unfortunately, these archived fingerprints can become challenging to analyse for a variety of reasons.

First and foremost, ALFPs are typically considered low template DNA samples, or samples with less than ∼100 pg of available DNA [10]. The amount of DNA left behind after an individual touches a surface is highly variable, ranging from fewer than 10 pg to upwards of 500 pg [11,12]. Latent prints can be challenging to process [10] because STR analysis works best when the amount of template DNA in the polymerase chain reaction (PCR) is between 0.5–2.0 ng [2,13–16]. Additionally, low template samples are more easily compromised *via* contamination, as they may only contain a few cells, meaning that only a few stray cells from an outside source could contribute as much DNA to the sample as the original contributor [2]. In a 2009 study of 252 low template DNA samples, only 8% yielded a full STR profile, while 6% yielded a partial profile with more than 12 alleles, and 44% resulted in no profile at all [17]. Results like this can cause crime laboratories to be reluctant to process these samples, even though they may contain valuable evidence.

Aside from the low template nature of fingerprint samples, there are many other variables that can impact success of DNA analysis in these samples. In fact, it is difficult to predict the amount of DNA found in touch samples, as there are a variety of individual factors that can influence the result. For example, individuals can be categorised as “good” or “bad” shedders of biological material [18]. Additionally, general habits of the person can contribute to the amount of shed DNA, such as frequent handwashing or habitual touching of the face or hair [6]. The surface upon which the fingerprint was deposited will also impact the amount of DNA that can be recovered. For example, non-porous surfaces, such as glass, tend to trap less cellular debris than porous ones, like fabric, and thus provide less DNA [12]. However, while porous substrates may retain more DNA, the uneven nature of the surfaces makes the development of latent fingerprints using chemicals or visualisation powders more difficult, and as such, fewer ALFP lift cards submitted to a forensic lab are from porous surfaces [4]. Unfortunately, many of these on-scene visualisation enhancements can greatly reduce the success of DNA profiling from fingerprints [9,19–21]. Lastly, removal of the cellular debris to another substrate, such as the tape-lifting method used for ALFP collection, may fail to collect 100% of available biological material [22] *versus* the use of more direct methods [11,17,22,23]. The best approach for latent fingerprint collection may be to collect surface swabs following a tape lift of the fingerprint. However, studies evaluating a combined sample of a surface swab with an ALFP have not yet been conducted.

While the variables discussed above create the greatest challenges to ALFP lift card sample DNA processing, the analytical protocols used in the lab for DNA analysis can also impact sample success. A study by Solomon et al. [9] examined biological evidence retrieval techniques, DNA extraction methods, and DNA concentration methods, and determined an optimised workflow for ALFP samples that would improve both DNA yields and STR profiles. Each variable was investigated individually to determine the optimal combination of steps to maximise the quality of the DNA profiles obtained. Most notably, in this study, only 20% of the DNA samples concentrated with Microcon^®^ Y-100 DNA Fast Flow Centrifugal Filter Units (Millipore, Billerica, MA, USA) resulted in detectable STR allele peaks, while 90% of those concentrated using Centri-Sep Spin Columns (Princeton Separations, Adelphia, NJ, USA) had detectable STR allele peaks [9]. This is significant as Microcon filters are one of the commonly used methods of DNA concentration in crime labs [10]. While the workflow elucidated in the Solomon et al. [9] study was shown to significantly improve DNA processing from ALFP samples, unfortunately, only one-month old fingerprints were tested, which may not be an accurate representation of the age of samples a laboratory would be processing with this workflow. Testing of these workflow modifications on older, more challenged ALFP lift cards is needed to fully evaluate the utility of the optimised workflow [24]. In this work, the viability of aged ALFP lift cards as a more realistic forensic-type sample was tested using the optimised workflow developed by Solomon et al. [9].

## Materials and methods

### Sample collection

Fingerprints from volunteers were collected on glass and glossy paper. Half of each individual’s set of fingerprints from each substrate were left untreated while the other half were visualised with magnetic powder (EVIDENT, Union Hall, VA, USA). For those, magnetic powder was dusted onto the fingerprints using a magnetic applicator. For collection of the fingerprints on glossy paper, fingerprint tape (EVIDENT) was placed directly onto the paper while fingerprints deposited on glass were tape-lifted and placed onto a 8 cm × 13 cm paper lift card (EVIDENT). Fingerprints were stored undisturbed in a separate enclosure at room temperature and processed at either 4 weeks post-collection (“fresh”) or 2 years post-collection (“aged”). An individual fingerprint from each substrate and each treatment type (untreated *vs*. magnetic power-treated) was processed using a traditional forensic DNA workflow described below; two of each were processed using the optimised workflow previously described [9] and described below. Use of thumb and pinky prints was avoided to reduce variation in DNA deposits due to inconsistent fingerprint sizes. A reference buccal swab was collected from each volunteer fingerprint donor; buccal swabs were subsequently used to develop reference STR profiles for comparison in each of the studies detailed below. All samples processed for this study were done so by the same examiner using the same reagents.

Additional mock-casework ALFP lift card samples collected at Virginia Commonwealth University (aged 5, 7, and 10 years, *n* = 7, 9, and 10, respectively) were used for these studies, along with non-probative “super-aged” samples donated from the Virginia Beach Police Department (aged 28 years, *n* = 10). For super-aged samples, seven 28-year-old, nine 10-year-old, ten 7-year-old, and ten 5-year-old samples were processed with the optimised workflow previously described [9] and described below.

### DNA extraction and concentration

#### Traditional workflow

For ALFP samples processed using a traditional forensic DNA workflow (including those lifted from glass and paper, *n* = 50), DNA was collected using the double swab technique [25]. Following disassembly of the ALFP lift card, the sides of the tape and paper containing cellular debris from the collected fingerprint were swabbed separately, first with a sterile swab (pre-wet with 150 µL 2% SDS), followed by a dry swab. The swabs taken from the tape side were placed in separate tubes than those from the paper side. All swabs were FisherBrand (Fisher Scientific, Hampton, NH, USA). All samples were lysed in 800 µL Buffer ATL (Qiagen, Germantown, MD, USA) and 40 µL Proteinase K (Qiagen) for 1 h at 56 °C. Forceps were used to remove swabs from the lysate, and all lysates were then transferred and combined over a single MinElute column (Qiagen) for DNA extraction using the QIAamp^®^ DNA Investigator Kit (Qiagen) as described by Solomon et al. [9]. After DNA quantification (described below), DNA samples were concentrated with Microcon^®^ Y-100 DNA filters. The entire DNA sample was added to the column along with 70 µL TE (10 mmol/L Tris, 100 µmol/L EDTA, pH 7.5), followed by a 13-min spin at 500× *g*. Next, 70 µL TE was added to the column to wash the DNA, which was then spun at 500× *g* for 14 min. For the final step, no TE was added and the filter was inverted and spun into a clean tube for 3 min at 1 000× *g*. This resulted in a final volume of approximately 5–8 µL. Lastly, ddH_2_O was added to bring the sample to a final concentration of 0.2 ng/µL. If less DNA was available or none was detected in the sample, no ddH_2_O was added and the final volume was left at 5–8 µL.

#### Optimised workflow

For the ALFP optimised workflow (including those lifted from glass and paper, *n* = 100), the paper and tape sides were disassembled and cut into small squares (approximately 3 mm × 3 mm in size). The tape and paper cuttings were then placed in separate tubes and lysed overnight at 56 °C in 300 µL Buffer ATL and 20 µL Proteinase K. The tape and paper were removed from the lysate, placed in a spin basket, placed back into the lysate tube, and centrifuged at 6 000× *g* for 1 min to remove any remaining liquid. The lysate then was pipetted onto a MinElute column and DNA was extracted using the QIAamp^®^ DNA Investigator Kit as described by Solomon et al. [9]. After quantification (described below), samples were further purified using Centri-Sep columns. The columns were rehydrated with 800 µL sterile ddH_2_O and incubated at room temperature for 2.5 h. The bottom of each column was then opened and excess water allowed to flow out. Columns were then spun at 800× *g* for 2 min. Last, DNA samples were loaded onto separate columns and spun again for 2 min at 800× *g*. After purification, the samples were concentrated using a Savant^TM^ DNA 120 SpeedVac^TM^ (Thermo Fisher Scientific, Waltham, MA, USA) vacuum centrifuge to obtain a final estimated concentration of 0.2 ng/µL, based on the known DNA concentration from the original quantification step. If less DNA was available or none was detected, samples were concentrated to a final total volume of 5–8 µL.

### DNA quantification, amplification, and analysis

DNA samples from all experiments were quantified using the Investigator^®^ Quantiplex Kit (Qiagen) on an ABI PRISM^®^ 7500 thermocycler using SDS v1.2.3 software (Applied Biosystems, Foster City, CA, USA) following the manufacturer’s protocol [26], modified for a half volume reaction. Samples with any amount of DNA measured during quantification (results other than “undetected”) were characterised as those with “detectable” DNA. DNA yields were calculated by multiplying the concentration by the elution volume, and a mean yield was calculated for each experimental group. STR amplification was achieved using AmpFℓSTR^®^ Identifiler^®^ Plus PCR Amplification Kit (Applied Biosystems) on a GeneAmp^®^ 9600 PCR System (PerkinElmer, Waltham, MA, USA) following the manufacturer’s protocol [27] modified for half volume reactions and a 45 min 60 °C final extension.

STR amplicons were analysed using an ABI PRISM^®^ 3130 Genetic Analyser (Applied Biosystems) with GeneMapper^®^ ID software v4.1 (Life Technologies, Carlsbad, CA, USA). For capillary electrophoresis, 3 µL amplified DNA was combined with 0.1 µL GeneScan™ 500 LIZ^®^ Size Standard (Life Technologies) and 10.5 µL Hi-Di formamide (Life Technologies). Sample analysis included 15 s, 3 kV injections with 25 min, 15.0 kV electrophoresis runs on a 36 cm capillary filled with POP-4^TM^ polymer (Applied Biosystems). All samples were analysed using a previously validated 50 RFU analytical threshold and a 250 RFU stochastic threshold. Data were compared to reference samples to determine the percent of expected STR peaks (i.e. those present in the reference profile) detected in each profile (Amelogenin was not included in data analysis). An average STR allele peak height was calculated for each sample (homozygous peak heights were halved). Known artifacts were excluded from calculations. Drop-in (unknown) alleles were counted. Differences between experimental groups were compared using a Students *t*-test (α = 0.05).

## Results and discussion

### Evaluation of optimised method on aged ALFP lift cards

**Figure 1.**
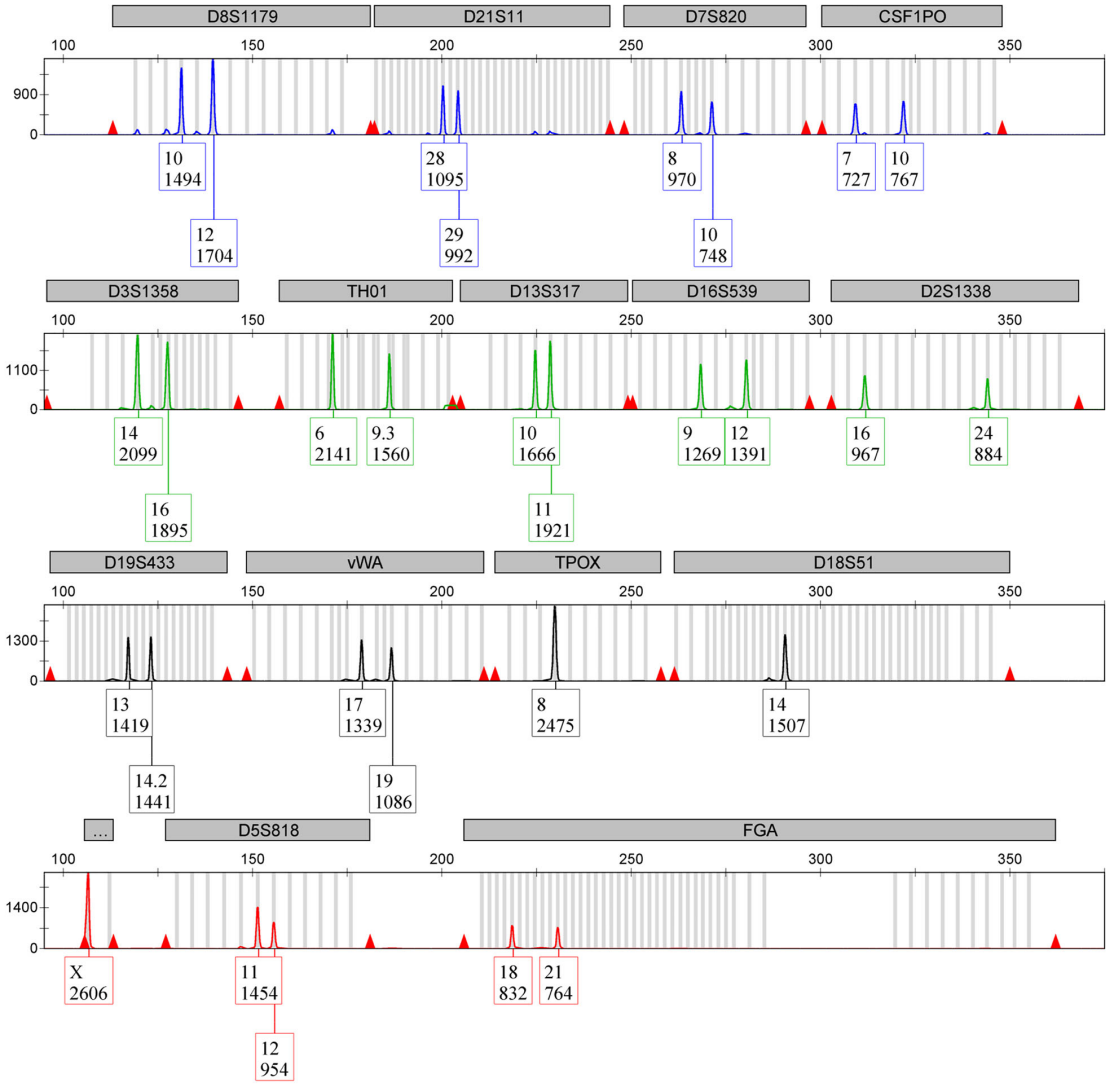
Short tandem repeat (STR) electropherogram of aged (2-year-old) archived latent fingerprint (ALFP) lift card DNA sample processed using the optimised workflow. This ALFP lift card sample was treated with magnetic powder prior to collection. The resulting STR profile is representative of the four total from this group that included detection of all expected STR alleles. All peaks are well above the stochastic threshold, with no artifacts noted.

Data obtained from ALFP lift cards processed with the optimised and traditional workflows were compared to determine if the advantages of the optimised workflow previously reported [9] hold true with more compromised ALFP lift card samples. Although fewer aged samples yielded detectable DNA and interpretable STR profiles than was reported when fresh samples were used [9], when the optimised workflow was used, increased DNA yields and improved STR profiles were observed. Aged ALFP samples treated with magnetic powder trended towards higher DNA yields and greater numbers of expected STR alleles (Table 1), including four samples that produced complete STR profiles (Figure 1). The insignificant *P-*values observed are not surprising, as it has been shown that low template samples can produce highly variable qPCR results [28]. Further, the differences observed in the STR profiles generated by the optimised workflow could be of great practical value as this represents an increase of approximately five reportable STR alleles, which could be enough to singlehandedly make a previously ineligible CODIS sample eligible. Alternatively, no differences were noted in either DNA yields or STR profiles from untreated ALFP lift card samples when the two workflows were compared (Tables 1 and 2). No differences in STR data quality, as measured by allele peak height, were noted between the two workflows, regardless of treatment (Table 1). This observation is consistent with the results from Solomon et al. [9], who noted that DNA yields and data quality from untreated samples were not impacted by the workflow used. Interestingly, the optimised workflow produced fewer samples with detected STR alleles (32.63% overall *versus* 47.92% from the traditional workflow, Table 1). However, the optimised method did trend towards recovering more STR alleles per sample, which would likely be a positive trade-off.

**Table 1.** Effect of DNA workflow on DNA yields and observed short tandem repeat (STR) profiles obtained from aged archived latent fingerprint (ALFP) samples.

DNA workflow	Samples with DNA detected (%)	Average DNA yield (ng)		Samples with detected STR alleles (%)	Expected STR alleles observed (%)		Average peak height^a^
		Magnetic^a^	Untreated^a^		Magnetic^a^	Untreated^a^	
Traditional (*n* = 47)	74.47	0.170 ± 0.359	0.276 ± 0.627	47.92	28.45 ± 34.24	36.89 ± 43.51	701.21 ± 703.08
Optimised (*n* = 95)	62.10	0.391 ± 0.970	0.251 ± 0.512	32.63	48.77 ± 40.32	33.24 ± 34.64	680.36 ± 797.71

^a^*P* > 0.05, no statistical significant.

**Table 2.** Effect of storage time on DNA yields obtained from archived latent fingerprint (ALFP) samples using an optimised workflow.

Age of ALFP	Samples with DNA detected (%)	Average DNA yield (ng)	
		Magnetic^a^	Untreated^a^
Fresh (*n* = 20)	95.00	0.667 ± 1.490	0.255 ± 0.298
Aged (*n* = 36)	69.44	0.918 ± 1.440	0.481 ± 0.715

^a^*P* > 0.05, no statistical significant.

**Figure 2.**
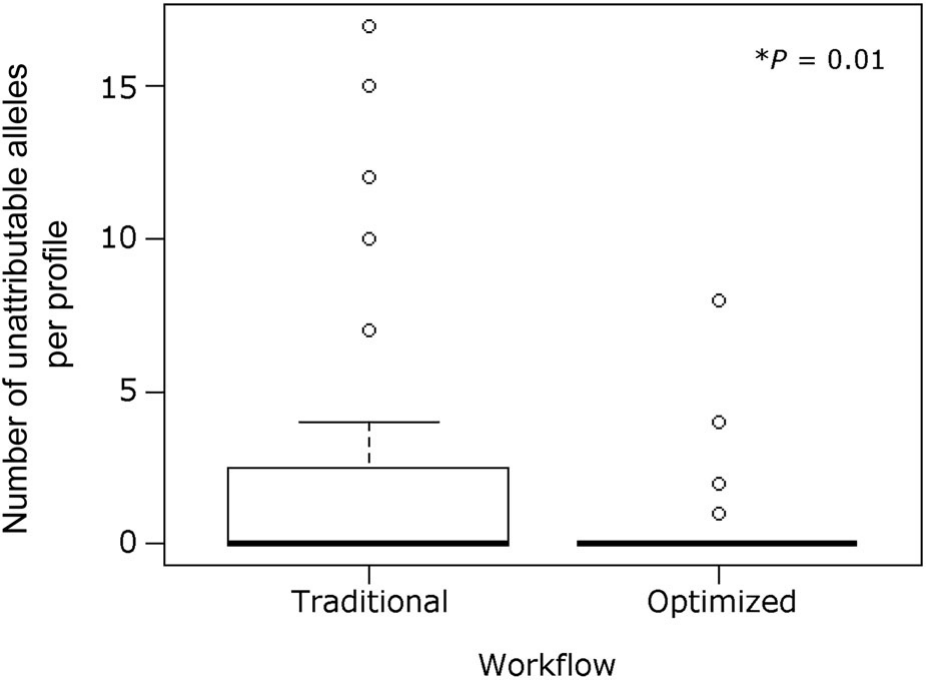
Number of unattributable short tandem repeat (STR) alleles observed in 2-year-aged samples for each workflow tested. While most samples analysed with either method showed no unattributable STR alleles (including drop-in alleles), those following the traditional workflow had significantly more unattributable alleles present (1.96 per profile, *n =* 47) compared to the optimised workflow (0.45 per profile, *n* = 95). This could be caused by variations in the workflow, such as sample preparation or concentration method used.

STR data from ALFP lift card samples were carefully evaluated for the presence of common STR artifacts, including pull-up, stutter, and incomplete adenylation. Artifacts were rarely observed in the STR data discussed herein, regardless of visualisation treatment (Figure 1). This was not surprising, as many common artifacts are more often associated with high amounts of template DNA [2]. The number of unattributable STR alleles were also counted for each ALFP lift card sample analysed [9,27]. Unexpected alleles, unattributable to laboratory staff or sample donors, were observed across all aged (2-year-old) ALFP lift card sample groups, regardless of treatment or workflow. No contamination was observed in any reagent blanks or negative PCR controls throughout the study. Notably, however, there was a significant reduction in the number of alleles not attributable to the donor profile observed when samples were processed using the optimised workflow *versus* a traditional workflow (Figure 2, *P*=0.01) despite a number of additional steps added to the extraction protocol of the optimised workflow. This indicates that a causative factor for this decrease is likely rooted in the differences between the traditional and optimised workflows. The primary differences between these workflows are the initial sample preparation/cell retrieval method and the method of sample concentration/re-purification. Regarding the collection method, the swabs and swab boxes used for traditional ALFP lift card processing and DNA collection, although they arrive pre-sterilised, are not sterilised in the lab, whereas the tools recommended for use with the optimised method to produce cuttings (namely forceps and scissors) are individually sterilised with bleach and ethanol just prior to use. This in-laboratory sterilisation may help to eliminate DNA from outside contributors that may persist on unsterilised materials as well as those that are purported to be DNA-free (a known issue in the forensic science community) [29]. As to the concentration and re-purification, the use of Centri-Sep columns with the reported optimised ALFP lift card processing seems superior to the Microcon method for removing impurities, as demonstrated by the increase in signal:noise ratio observed in the capillary electrophoresis data reported [9]. This likely lessens the impact of any minor DNA contributions that may be present in the ALFP lift card DNA samples. It cannot be stated whether one of these steps impacted the presence of unattributable alleles more than the other, or if it is the totality of the differences that caused the decreased number of drop-in when the optimised workflow was used.

### Impact of time on ALFP lift card analysis

To determine the effect of archival time on ALFP sample STR analysis success, ALFP lift card samples aged for 2 years (“aged”) were compared to those that were processed within 4 weeks of collection (“fresh”) using the optimised ALFP lift card workflow described previously [9]. Not unexpectedly, aged samples were 26% less likely to provide detectable DNA yields than fresh samples (Table 2) and those with detectable DNA were 34% less likely to result in a profile with any STR alleles peaks present (Table 3). However, interestingly, when DNA was detected from the aged samples, DNA yields were not significantly different from those noted from fresh ALFP lift card samples, regardless of visualisation treatment (Table 2, *P* ≥ 0.34 for all comparisons made). Both fresh and aged ALFP lift card samples provided STR profiles with ∼53% of the expected STR alleles detected above analytical threshold and mean STR allele peak heights above 900 RFU, showing that the overall STR profile quality, of the profiles obtained, was not impacted by age (Table 3). The data obtained showed that while fewer aged ALFP lift card samples resulted in STR profiles, the profiles obtained were on average equally as informative. Furthermore, as previously reported [9], fingerprints treated with magnetic powder prior to lifting provided more DNA than those left untreated, and this trend was not impacted by storage time (age) (Table 2). This is highly beneficial for the application of this workflow to ALFP lift card sample processing, as magnetic powder is commonly used at crime scenes, and it is unlikely that fingerprints left untreated would be frequently collected and stored.

**Table 3.** Effect of storage time on observed short tandem repeat (STR) profiles obtained from archived latent fingerprint (ALFP) samples using an optimised workflow.

Age of ALFP	Samples with detected alleles (%)	Expected STR alleles observed (%)^a^	Average Peak Height^a^
Fresh (*n* = 10)	90.00	53.52 ± 36.44	911.72 ± 778.63
Aged (*n* = 18)	55.56	53.29 ± 39.54	993.61 ± 907.71

^a^*P* > 0.05, no statistical significant.

**Figure 3.**
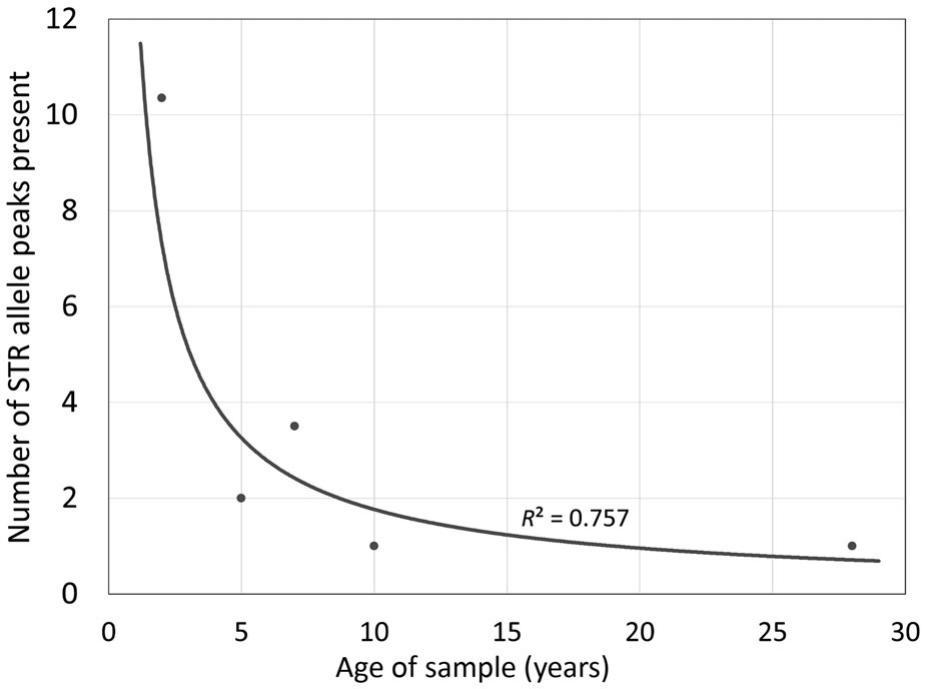
Effect of storage time on average number of alleles observed across entire short tandem repeat (STR) profiles from archived latent fingerprint (ALFP) samples using an optimised workflow. While a decrease in the number of detected alleles is observed as sample storage time increases, amplifiable DNA (DNA capable of being detected *via* capillary electrophoresis) was shown to be obtainable from all samples regardless of age.

In order to provide a more realistic understanding of the impact of the DNA workflow on ALFP lift card samples, “super-aged” samples were evaluated using the workflow previously optimised for ALFP samples. Super-aged samples used in this study were mock and/or non-probative casework samples that were collected in uncontrolled conditions ∼5–28 years ago. Detectable DNA was observed in half of all samples tested. Of the samples with detectable DNA, 5, 7, 10, and 28-year-old samples yielded an average of 0.088, 0.024, 0.006, and 0.100 ng of total DNA, respectively. As the archival age increased, there was a decrease in the number of alleles detected (Figure 3, *R*^2^=0.757). However, it is important to note that, of the 5-year-old ALFP lift cards tested, 40.0% had alleles present, as did 20.0% of 7-year-old, 12.5% of 10-year-old, and 14.3% of 28-year-old samples. While the STR profiles obtained from these super-aged ALFP samples were limited and may not be sufficient for identification/individualisation, the data are sufficient to be useful for exclusionary purposes, making it beneficial for these samples to be considered for DNA processing. Suspected individuals can easily be excluded as the contributor of an evidence sample profile with 1–2 loci that are exclusionary, making even limited STR data quite valuable.

## Conclusion

As DNA methods become increasingly more sensitive, old, degraded, or otherwise highly compromised biological evidence samples become more viable as options for human identification investigations. This includes the possibility of analysis from surface swabs from areas contacted or touched by a perpetrator, including those surfaces onto which latent fingerprints may have been deposited. Unfortunately, it was not possible to predict these technological advances 30 or more years ago, when DNA science was in its infancy. Consequently, swabbing surfaces for “touch” DNA was unheard of prior to 1997 [23], and fingerprints were more likely to have been preserved as “archived” latent fingerprint lift card samples — latents whose ridge detail has been visualised and tape-lifted for long term room-temperature storage on paper backing cards. While these conditions are not ideal for DNA retrieval and processing, the use of tape lifting does offer a secure way to isolate and store fingerprints. Furthermore, with the volume of cold or appellate cases that are now being revisited, ALFP lift card samples may be the only source of biological material available. Unfortunately, these samples offer many unique biological and technical challenges that routine DNA procedures do not adequately address. To this end, Solomon et al. [9] recently specified a series of sample-specific protocols that can be used in the DNA analysis workflow to improve success rates associated with the processing of ALFP lift card samples. However, the experiments performed in those studies were conducted using fresh ALFP lift card samples exclusively, rather than aged samples which are more representative of forensic casework needs.

The data described herein support the original findings of Solomon et al. [9], as the use of the optimised workflow on aged ALFP lift card samples (aged 2–28 years) often improved the number of STR alleles recovered, providing more discriminatory STR profiles than those processed using the traditional workflow. We further report that interpretable peaks can be detected from ALFP lift card samples stored as long as 28 years when the optimised procedures are followed. Furthermore, the exclusive use of individual laboratory-sterilised tools for sample preparation (without the use of intermediary substrates) and the addition of a re-purification step with Centri-Sep columns after DNA extraction in the recommended optimised workflow seem to limit the ability to detect low-level secondary DNA sources. This is a meaningful finding, as secondary DNA sources are more likely to be present in samples from older cases, when the use of personal protective equipment (PPE) and other precautionary measures were less common. Future work on ALFP lift card samples should explore the originating source of the unattributable/drop-in alleles noted in these samples in order to determine if they are more likely introduced from the collection materials, on-scene PPE, or from the fingerprint powders and tools commonly used. This information would allow for additional informed adjustments to the crime scene procedures used in an increasingly DNA-centered forensic system.
